# Supplementary material for: SHIFTR enables the unbiased identification of proteins bound to specific RNA regions in live cells
Source: Nucleic Acids Res. 2024 Jan 28;52(5):e26. doi: 10.1093/nar/gkae038 (PMC10954451; doi:10.1093/nar/gkae038)
Supplement: gkae038_Supplemental_Files [file gkae038_supplemental_files.zip › SUPPLEMENTARY FILES.pdf]

SUPPLEMENTARY FIGURES

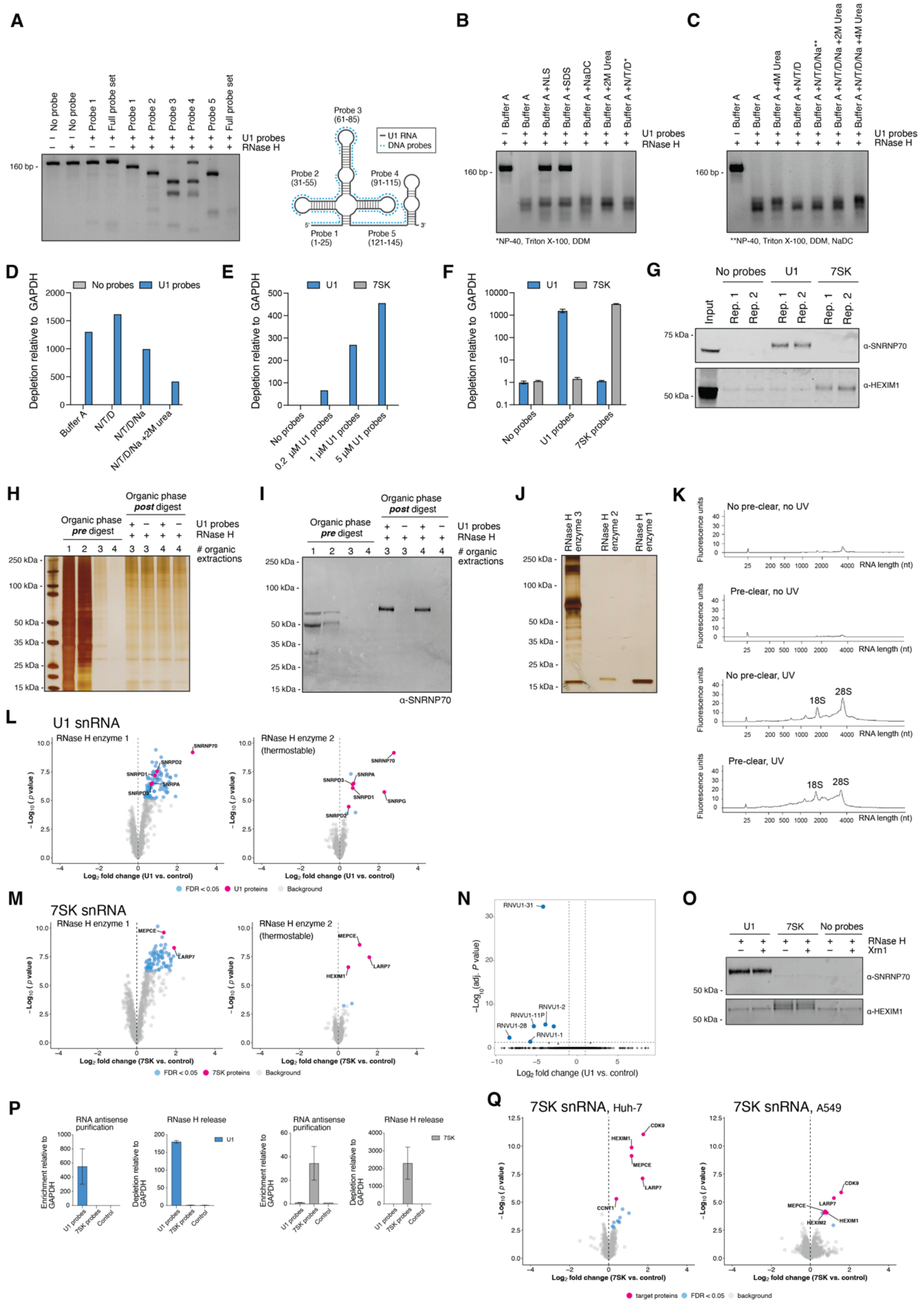

Figure S1: Optimization of experimental parameters for the effective digestion of target RNAs and the release of bound proteins from crosslinked interphases

(A) Gel electrophoresis analysis of *in vitro* transcribed U1 RNA and the cleavage patterns observed with DNA probes targeting different RNA regions as illustrated in schematic shown on the right. **(B)** Gel electrophoresis analysis of *in vitro* transcribed U1 RNA and its digestion by RNase H using sequence-specific DNA probes in buffers supplemented with different detergents and chaotropic agents as indicated. Buffer A: 50 mM Tris-HCl, 75 mM KCl, 3 mM $MgCl_2$, 10 mM DTT. **(C)** As in **(B)**, but supplementing digestion buffer with additional detergents and/or chaotropic agents. **(D)** RT-qPCR analysis of RNase H-mediated depletion of endogenous U1 RNA in digestion buffers supplemented with different detergents and chaotropic agents analyzed in (A) and (B). Interphases from UV-crosslinked Huh-7 cells were used. Depletion is normalized to GAPDH and compared to samples treated with RNase H in the absence of DNA probes (No probes). **(E)** RT-qPCR analysis of RNase H-mediated depletion of endogenous U1 RNA using different concentrations of DNA probes. Interphases from UV-crosslinked Huh-7 cells were used. Depletion is normalized relative to GAPDH. Unspecific cleavage is estimated by measuring changes to 7SK RNA levels. **(F)** RT-qPCR analysis of RNase H-mediated depletion of the endogenous U1 or 7SK snRNA in interphases isolated from UV-crosslinked cells. Depletion is normalized relative to GAPDH. Values are mean $\pm$ standard error of the mean (n = 2). **(G)** Western blot analysis of proteins released from UV-crosslinked interphases after the targeted digestion of U1 or 7SK snRNAs using sequence-specific DNA probes together with RNase H. SNRNP70 and HEXIM1 serve as positive controls for the U1 and 7SK snRNP complexes, respectively. Two independent replicates were analyzed. **(H)** Silver staining of SDS polyacrylamide gel analyzing the protein content of organic phases after several consecutive phase extraction steps before and after RNase H treatment of interphases with and without U1-specific DNA probes. **(I)** Western blot analysis of protein content of organic phases after several consecutive phase extraction steps before and after RNase H treatment of interphases with and without U1-specific DNA probes. SNRNP70 serves as positive controls for the release of U1 snRNP complex components. **(J)** Silver staining of SDS polyacrylamide gel analyzing the protein content of several different commercially available RNase H enzyme preparations. **(K)** Electropherogram of capillary electrophoresis analysis of RNA isolated from UV-crosslinked or uncrosslinked interphases with and without pre-clearing after protein removal by Proteinase K treatment. **(L)** Quantitative comparison of proteins released from UV-crosslinked interphases when using different commercially available RNase H enzymes for SHIFTR experiments targeting the U1 snRNA. SHIFTR was performed with pre-clearing. Volcano plots display $\log_2$ fold changes comparing U1-depleted to untreated samples. Known components of the U1 snRNP complex are highlighted in pink. Mass spectrometry experiments were performed without offline fractionation (Methods). **(M)** As in **(L)**, but for the 7SK snRNA. Known components of the 7SK snRNP complex are highlighted in pink. **(N)** Differential gene expression analysis of UV-crosslinked interphases after U1 degradation with thermostable RNase H and sequence-specific DNA probes compared to interphases subjected to treatment without the addition of probes and enzyme (control). Differentially expressed genes are highlighted in blue. **(O)** Western blot analysis of proteins released from UV-crosslinked interphases after the targeted digestion of U1 or 7SK snRNAs using sequence-specific DNA probes together with RNase H. RNase H treatment was followed with a 1 h Xrn1 exoribonuclease treatment as indicated. SNRNP70 and HEXIM1 serve as positive controls for the U1 and 7SK snRNP complexes, respectively. **(P)** RT-qPCR analysis

of the enrichment or depletion of the U1 or 7SK snRNAs from UV-crosslinked interphases using either RNA antisense purification, or RNase H-mediated target RNA depletion (RNase H release) as implemented in RAP-MS or SHIFTR, respectively. Enrichment and depletion of target RNAs is normalized relative to GAPDH. Values are mean ± standard error of the mean (n = 2). **(Q)** Deep proteome profile of 7SK SHIFTR experiments in Huh-7 (left) and A549 (right) cells using offline high pH reverse phase fractionation. Experiments shown correspond to data presented in Figures 1 E and G.

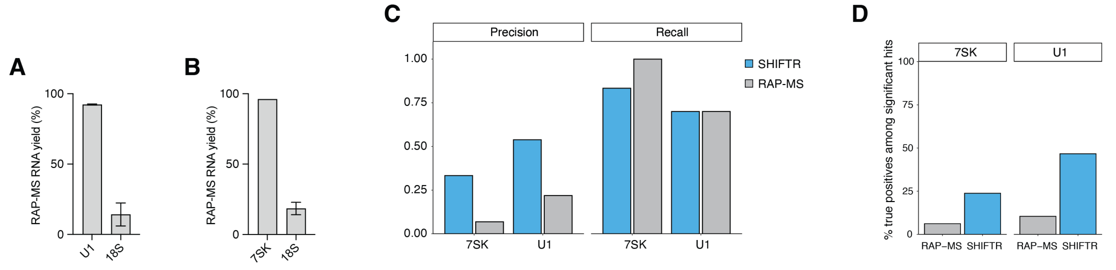

Figure S2: Side-by-side performance evaluation of SHIFTR and RAP-MS for identifying known U1 and 7SK components

(A) RT-qPCR analysis of RNA yield in RAP-MS experiments targeting the U1 snRNA. 18S rRNA serves as control. Yield is calculated relative to input. Values are mean ± standard error of the mean (n = 2). **(B)** As in **(A)**, but for the 7SK snRNA. **(C)** Comparison of precision and recall statistics for uncovering known components of the U1 and 7SK snRNPs using either SHIFTR (blue) or RAP-MS (grey) to identify directly bound proteins. **(D)** Analysis of the percentage of true positive hits (known U1 or 7SK components) among all significantly enriched proteins in SHIFTR and RAP-MS experiments targeting the U1 and 7SK snRNPs.

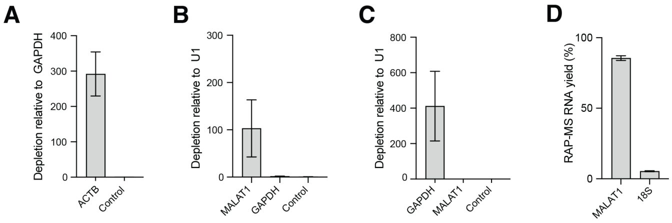

Figure S3: Targeting coding and non-coding RNAs with SHIFTR

(A) RT-qPCR analysis of RNase H-mediated depletion of the endogenous ACTB mRNA from UV-crosslinked interphases using sequence-specific DNA probes. Depletion is normalized relative to GAPDH. Interphase subjected to treatment without the addition of probes and enzyme serves as control. Values are mean ± standard error of the mean (n =2). Samples used for mass spectrometry analysis are shown. **(B)** As in **(A)**, but targeting the MALAT1 lncRNA. Depletion is normalized relative to U1. Interphases subjected to treatment with GAPDH-specific probes as well as interphases subjected to treatment without the addition of probes and enzyme serve as controls (n = 2, except for MALAT1 n= 4). **(C)** As in **(B)**, but targeting the GAPDH mRNA. Interphases subjected to treatment with MALAT1-specific probes as well as interphases subjected to treatment without the addition of probes and enzyme serve as control (n = 2). **(D)** RT-qPCR analysis of RNA yield in RAP-MS experiments targeting the MALAT1 lncRNA. Yield is calculated relative to input. 18S rRNA serves as control. Values are mean ± standard error of the mean (n = 2). Samples used for mass spectrometry analysis are shown.

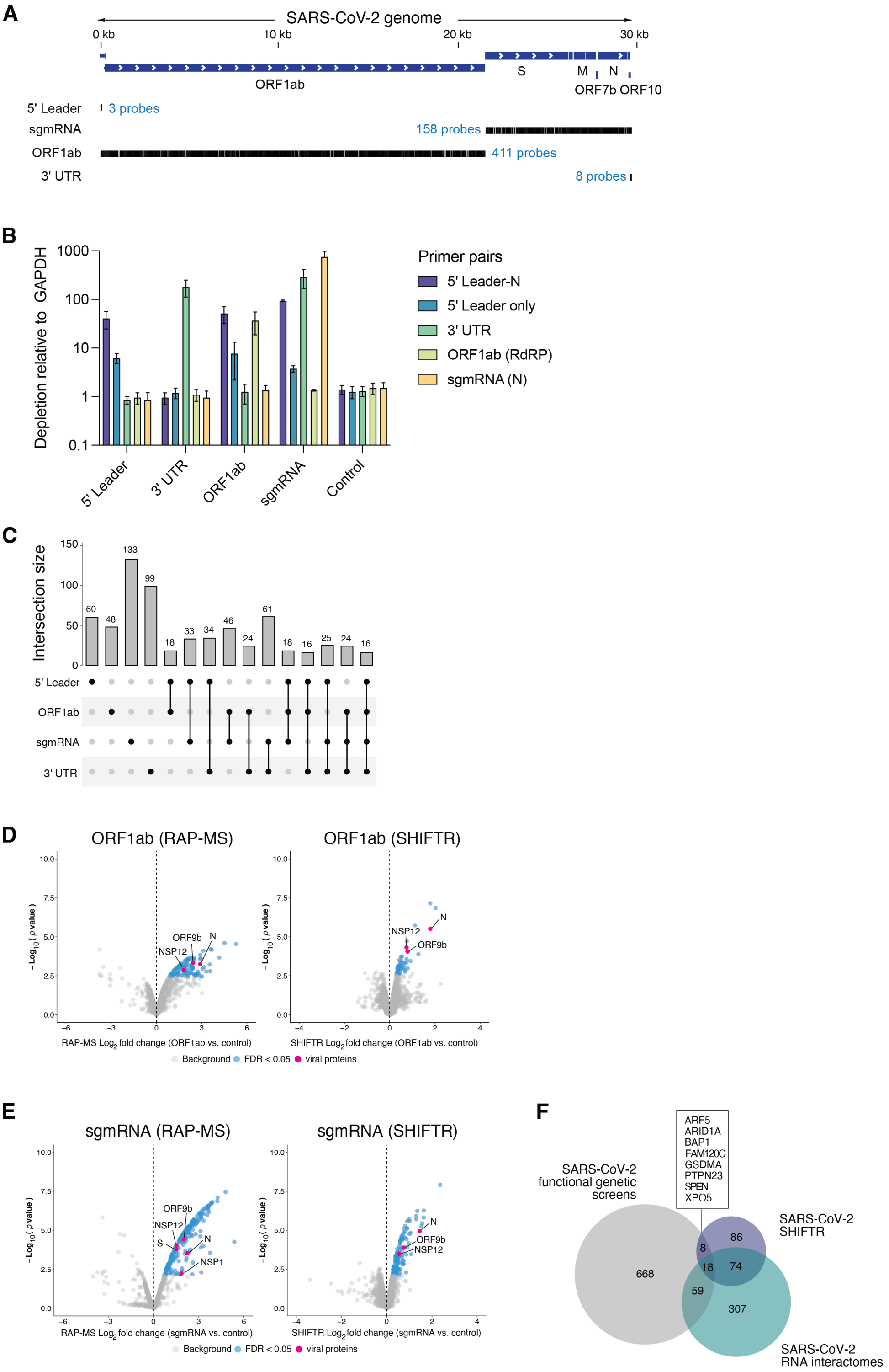

Figure S4: Interrogating different sequence regions in the authentic SARS-CoV-2 RNA genome with SHIFTR and RAP-MS

(A) Position of DNA antisense probes designed to hybridize to specific SARS-CoV-2 RNA regions to enable RNase H-mediated degradation of specific regions in viral RNA **(B)** RT-qPCR analysis of RNase H-mediated depletion of different regions in the SARS-CoV-2 RNA genome from UV-crosslinked interphases using sequence-specific DNA probes. Interphases

isolated from infected human A549[ACE2] cells (24 hpi) were used. Primer pairs targeting the indicated viral RNA regions (5′ leader-N, 5′ leader only, ORF1ab (RdRP), sgmRNA (N), 3′ UTR) were used and are indicated in different colors. Interphases treated with sequence-specific DNA probes are indicated on the x-axis. Samples subjected to treatment without the addition of probes and enzyme serve as control. Depletion is normalized relative to GAPDH. Values are mean ± standard error of the mean (n = 2). **(C)** Upset plot comparing the enrichment of proteins in SHIFTR experiment targeting different SARS-CoV-2 RNA regions. **(D)** Left: Quantitative analysis of proteins significantly enriched in purifications of SARS-CoV-2 RNA genomes using RAP-MS and targeting the ORF1ab region. Right: Quantitative analysis of proteins significantly enriched in SHIFTR experiments targeting the ORF1ab region in the SARS-CoV-2 RNA genome. Experiments were carried out in infected human cells at 24 hpi. Volcano plots display log_2 fold changes and significance estimates for identified proteins. RAP-MS experiments utilize the unrelated RNA RMRP as the control. SHIFTR experiments utilize UV-crosslinked interphases subjected to treatment without the addition of probes and enzyme as the control. Significantly enriched proteins are highlighted in blue, viral proteins are highlighted in pink. **(E)** As in **(D)**, but for SARS-CoV-2 sgmRNAs. RAP-MS experiments were first depleted for full length RNA genomes by capturing RNAs containing the ORF1ab region, prior to antisense-mediated capture of all sgmRNAs. **(F)** Venn diagram comparing hits identified in SARS-CoV-2 functional genetic screens (21 independent studies) (41) and SARS-CoV-2 RNA interactomes (5 independent studies) (41) with proteins significantly enriched in SHIFTR experiments targeting different SARS-CoV-2 RNA regions (5′ leader, ORF1ab, sgmRNAs, 3′ UTR).

SUPPLEMENTARY TABLE LEGENDS

Supplementary Table S1: Quantitative mass spectrometry data related to Figure 1. SHIFTR optimization experiments targeting the endogenous U1 and 7SK snRNAs in two different cell types. Control data (no enzyme, no probe) is included in table as well.

Supplementary Table S2: Quantitative mass spectrometry data related to Supplementary Figure S1. SHIFTR optimization experiments recorded without offline high pH reverse phase fractionation. Control data (no enzyme, no probe) is included in table as well.

Supplementary Table S3: Quantitative mass spectrometry data related to Figure 2 and 3. Side-by-side comparison between SHIFTR and RAP-MS for different endogenous target RNAs.

Supplementary Table S4: Quantitative mass spectrometry data related to Figure 3. Multiplexed SHIFTR experiments targeting the endogenous ACTB mRNA and MALAT1 lncRNA are shown together with individual SHIFTR experiments targeting the GAPDH mRNA.

Supplementary Table S5: Quantitative mass spectrometry related to Figure 4. SHIFTR experiments targeting different sequence regions in the SARS-CoV-2 RNA genome (5′ leader, ORF1ab, sgmRNAs, 3′ UTR) are shown. GO enrichment analyses for each target RNA interactome (biological process (BP) and molecular function (MF)) is provided in separate worksheet.

Supplementary Table S6: Oligonucleotide sequences used in this study.
